# Intravenous Ibuprofen Versus Ketorolac for Perioperative Pain Control in Patients with Morbid Obesity Undergoing Bariatric Surgery: A Randomized Controlled Trial

**DOI:** 10.1007/s11695-025-07752-5

**Published:** 2025-03-10

**Authors:** Sarah Amin, Ahmed Hasanin, Suzy Soliman, Maha Mostafa, Ahmed S. Abdallah, Dina Zakaria, Amr Abdelkader

**Affiliations:** Cairo University, Giza, Egypt

**Keywords:** Bariatric surgery, Obesity, Ketorolac, Ibuprofen, Nalbuphine, Visual analogue scale

## Abstract

**Background:**

We aimed to compare the perioperative analgesic efficacy of intravenous ibuprofen versus ketorolac in patients with obesity undergoing bariatric surgery.

**Methods:**

This randomized controlled trial included adult patients with obesity undergoing bariatric surgery. Participants were randomized to receive either ibuprofen or ketorolac intravenously every 8 h. All patients received paracetamol intravenously 1 gm/6 h. Inadequate intraoperative analgesia was managed by fentanyl boluses, while inadequate postoperative analgesia was managed by nalbuphine boluses. The primary outcome was static visual analogue scale (VAS) 0.5 h postoperatively. Secondary outcomes were postoperative static and dynamic VAS, intra- and postoperative opioids consumption, postoperative nausea and vomiting, and patients’ satisfaction.

**Results:**

Fifty-three patients were analyzed in each group. The median VAS (quartiles) at 0.5 h postoperatively was lower in the ketorolac group (3 [3, 6]) than in the ibuprofen group (7 [4, 8]), *P*-value < 0.001. The static and dynamic VAS were lower in the ketorolac group than in the ibuprofen group up to 6 h postoperatively. The intra- and postoperative opioid consumption was lower in the ketorolac group than in the ibuprofen group. The incidence of postoperative nausea and vomiting was also lower in the ketorolac group than in the ibuprofen group. Patients in the ketorolac group had higher level of satisfaction than patients in the ibuprofen group.

**Conclusion:**

In patients with obesity undergoing bariatric surgery, perioperative administration of ketorolac provided improved pain control, reduced opioid consumption, and lowered the risk of postoperative nausea and vomiting, compared to ibuprofen. Additionally, patients reported higher satisfaction with ketorolac.

**Supplementary Information:**

The online version contains supplementary material available at 10.1007/s11695-025-07752-5.

## Introduction

Bariatric surgery is a common surgical procedure, and it is recommended for effective treatment for obesity and its related morbidities. Perioperative pain management after bariatric surgery is essential to allow early ambulation, reduce pulmonary complications, and facilitate early discharge from the hospital [1]. Using a multimodal approach for pain management is common in most centers; however, in patients with obesity, the analgesic choices are relatively limited due to several factors [1, 2]. Opioid drugs have many side effects such as respiratory depression and subsequent airway obstruction, nausea, vomiting, physical dependence, and hormonal and immunologic dysfunction [3]. Furthermore, the anatomical difficulties make regional blocks challenging [4, 5]. Thus, the use of parenteral non-opioid drugs for analgesia is important to provide the highest possible pain relief whether they were used solely or in combination with other modes of analgesia.

Ketorolac and ibuprofen are two commonly used non-steroidal anti-inflammatory drugs and can be administered intravenously; thus, both drugs can be conveniently administered in the perioperative period. The two drugs had been previously used in bariatric surgery with good results [6–9]. However, to the best of our knowledge, no studies had compared the two drugs in bariatric surgery. The aim of the current study was to compare the analgesic effects of both drugs in patients with obesity undergoing bariatric surgery.

## Methods

This randomized, double-blinded controlled trial was conducted at a university hospital between April 2023 and January 2024, following approval from the institutional ethics committee (MS-340–2022) and registration at clinicaltrials.gov. Written informed consent was obtained from all participants prior to enrollment.

Participants were adult patients aged 18 to 65 years with obesity (body mass index > 35 kg/m^2^) and an American Society of Anesthesiology (ASA) physical status of II-III scheduled for laparoscopic bariatric surgery.

Exclusion criteria were history of ischemic heart disease, known obstructive sleep apnea or patients with STOP-bang score ≥ 5, baseline SpO_2_ < 95%, renal impairment, allergy to any of study’s drugs, history of gastrointestinal bleeding or ulceration, or inflammatory bowel disease.

Group randomization into a 1:1 ratio was performed using an online resource (https://www.graphpad.com/quickcalcs/randomize1/). The randomization sequence, group assignment, and drug preparation instructions were placed inside sequentially-numbered opaque envelopes. A research assistant handled envelope opening, group assignment, and drug preparation without any further involvement in the study. The three doses were prepared beforehand and marked with the patient’s name, hospital number, and time of administration. The prepared doses were stored in a refrigerator. The attending nurse was instructed to give the drug according to the written time on the prepared drug. The patient, attending anesthetist, nurse, and data collector were blinded to the administered drug.

All patients received 1 g of intravenous paracetamol 30 min before surgery, then every 6 h postoperatively. In addition, patients in the ketorolac group received 30 mg of intravenous ketorolac (Ketolac 30 mg/ 2 mL, AMRIYA PHARM. IND, Alexandria, Egypt), while patients in the ibuprofen group received 800 mg of intravenous ibuprofen (Ibuprofen-Arabcomed 100 mg/mL, ARABCOMED, Cairo, Egypt). Both drugs were diluted in 200 mL of normal saline and infused over 5 min. Postoperatively, both drugs were given every 8 h.

Upon arrival to the operating room, routine monitors (non-invasive blood pressure monitor, electrocardiogram, and pulse oximetry) were attached, and 5 mg dexamethasone was given slowly after securing intravascular access.

General anesthesia was induced with 2 mg/kg propofol, 2 mcg/kg fentanyl (lean body weight), and atracurium 0.5 mg/kg (ideal body weight). Anesthesia was maintained with isoflurane 1–1.2% in oxygen/air admixture and 0.1 mg/kg atracurium every 30 min. Systolic blood pressure and heart rate were recorded every 15 min during the procedure. A fentanyl bolus of 1 mcg/kg was given if the heart rate and/or systolic blood pressure were > 120% of baseline reading in the absence of other causes.

Static and dynamic visual analogue scale (VAS) were assessed at rest and during cough at 0.5, 2, 4, 6, 10, 18, and 24 h postoperatively. If the VAS score exceeded 3, intravenous nalbuphine at a dose of 0.1 mg/kg (based on lean body weight) was administered, titrated to the patient’s response, with a maximum single dose of 20 mg and a maximum daily dose of 160 mg. Systolic blood pressure and heart rate were recorded at the same time points as the VAS.

In case of postoperative nausea or vomiting, intravenous ondansetron 4 mg was given.

At 24 h postoperatively, the patients were asked to rate their level of satisfaction with pain control using a 0-to-10 scale, where a score of 0 indicated strong dissatisfaction and a score of 10 indicated strong satisfaction.

The primary outcome was Static VAS 0.5 h postoperatively. Secondary outcomes were static and dynamic VAS, intraoperative fentanyl consumption, postoperative nalbuphine consumption, time to first postoperative nalbuphine request (defined as the time from extubation to the first analgesic request), time to independent movement (defined as the time from extubation to the ability to move independently), ability to pass flatus within the first 24 h postoperatively, postoperative nausea, vomiting, itching, level of sedation using the modified Ramsay sedation scale, respiratory depression (defined as a respiratory rate less than 8 breath per min), and satisfaction level. Other outcomes included perioperative systolic blood pressure and heart rate, duration and type of surgery, age, weight, body mass index, and comorbidity.

### Statistical Analysis

In a previous study [9], static VAS in the post-anesthesia care unit in patients who received intravenous ibuprofen was 3.4 ± 1.24. We calculated the sample size of the current study to detect a 20% difference between the VAS in the two study groups. For a study power of 80% and alpha error of 0.05, the minimum number of patients needed in each group was 106 (53 patients per group). The number of envelopes was increased to 116 to compensate for possible dropouts. The sample size was calculated using MedCalc (14.10.2) software.

Statistical package for social science (SPSS) software, version 26 for Microsoft Windows (IBM. Corp., NY, USA), was used for data analysis. Categorical data are presented as frequencies (%) and were analyzed using the Chi-square test or Fisher’s exact test, as appropriate. Continuous data were assessed for normality using the Shapiro–Wilk test and are presented as mean ± standard deviation or median (quartiles), depending on the data distribution. Unpaired continuous data were analyzed using either the unpaired* t*-test or the Mann–Whitney test, based on normality. The time to the first nalbuphine request was compared using the log-rank test. Repeated measures data were analyzed using repeated measures analysis of variance to determine within-group and between-group differences. The Bonferroni correction was applied to adjust for multiple testing, and adjusted *P*-values were used to determine significance. Static and dynamic VAS scores were analyzed using the generalized estimating equation (GEE) model, which included the main effects of group, time, and total nalbuphine dose. A *P*-value of less than 0.05 was considered statistically significant.

## Results

**Fig. 1:**
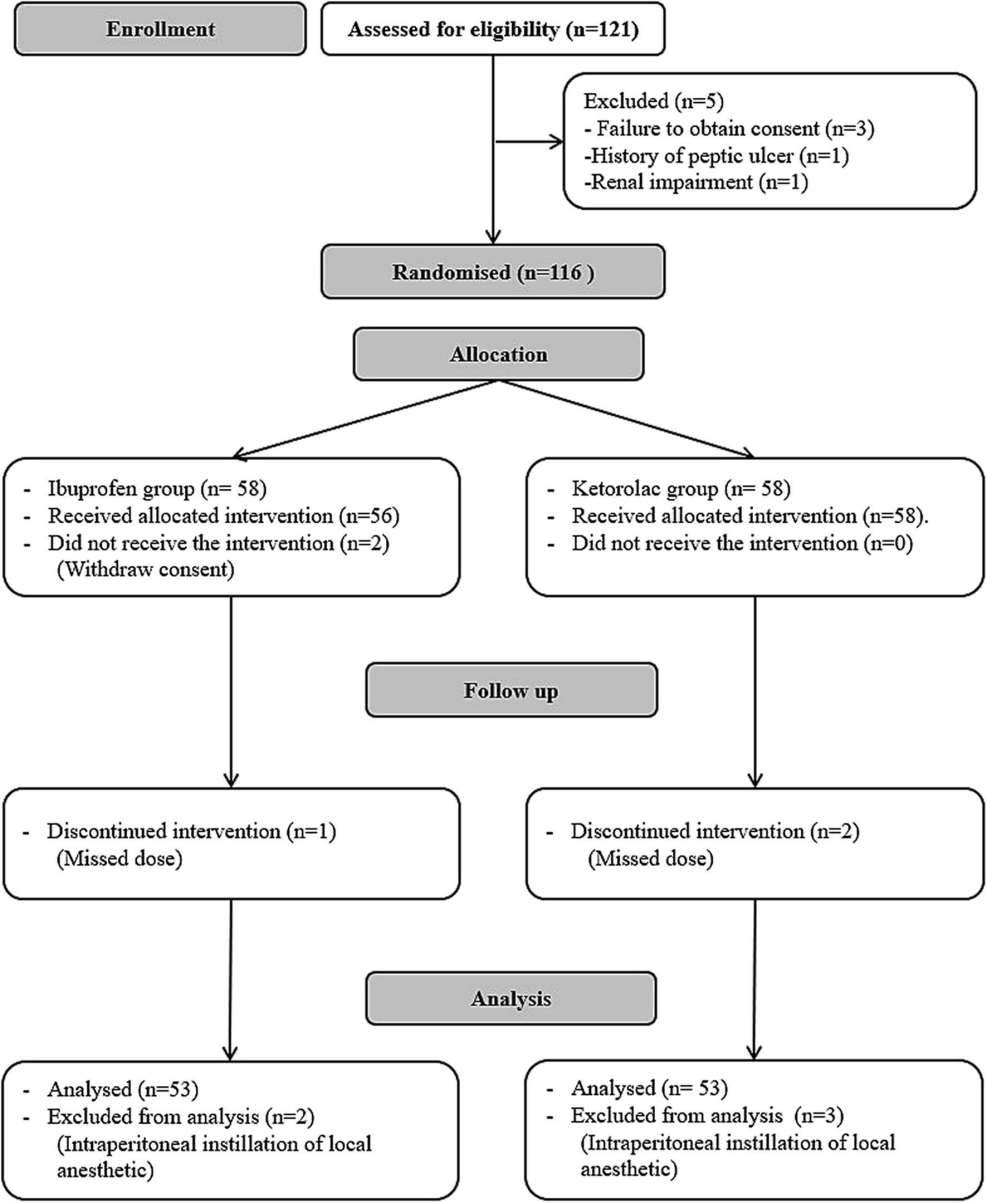
CONSORT’s flowchart

One hundred and twenty-one patients were screened for eligibility, five patients were excluded for not meeting the inclusion criteria, and 116 patients were randomized into one of the study’s groups. Five patients in each group were excluded, and 53 patients in each group were analyzed (Fig. 1).

Demographic data and surgical characteristics were comparable between the two groups (Table 1). All procedures were primary bariatric surgeries, not revision surgeries. Only one patient in the ibuprofen group underwent cholecystectomy in addition to sleeve gastrectomy. None of the participants had a significant surgical history.

**Table 1:** Demographic data. Data are presented as mean ± standard deviation, median (quartiles), and frequency (%)

	Ibuprofen group (*n* = 53)	Ketorolac group (*n* = 53)	*P*-value
Age (years)	34 ± 10	36 ± 9	0.183
Male sex	7 (13%)	16 (30%)	0.058
Weight (kg)	130 (120, 140)	135 (119, 141)	0.997
Body mass index (kg.m^−2^)	49 (47, 55)	48 (45, 54)	0.566
ASA-PS			0.604
II	52 (98%)	51 (96%)	
III	1 (2%)	2 (4%)	
Comorbidity Hypertension Diabetes mellitus	12 (23%) 9 (17%)	10 (19%) 9 (17%)	0.632 1.000
Type of procedure Sleeve gastrectomy Gastric bypass	50 (94%) 3 (6%)	49 (93%) 4 (8%)	0.696
Duration of the procedure (min)	120 (90, 165)	138 (120, 165)	0.446

*ASA-PS*, American Society of Anesthesiologist-physical status

**Fig. 2:**
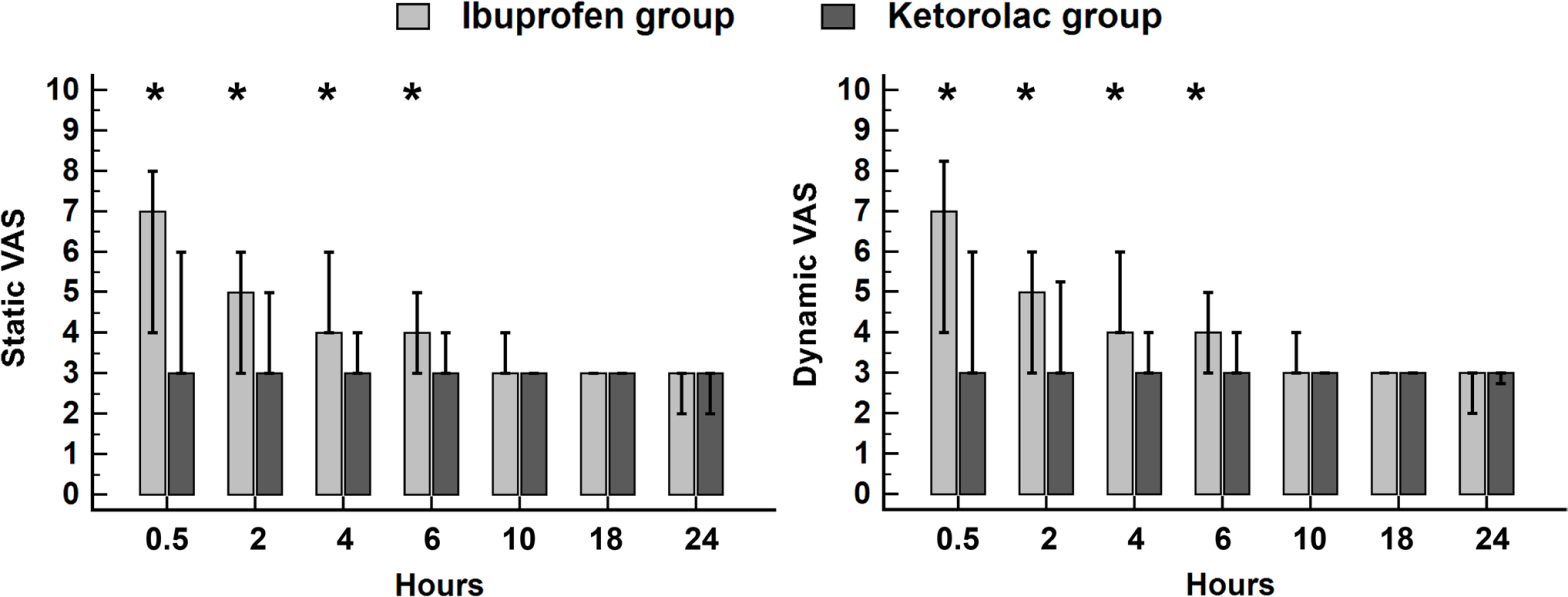
Bar and whiskers plot for postoperative static and dynamic VAS. Bars represent the median, and whiskers represent the 25th and 75th percentiles. * Denotes statistical significance between the groups. VAS, visual analogue scale

The median VAS (quartiles) at 0.5 h postoperatively was 3 (3, 6) in the ketorolac group and 7 (4, 8) in the ibuprofen group, *P*-value < 0.001 (Table 2). The static and dynamic VAS was lower in the ketorolac group than in the ibuprofen group up to 6 h postoperatively (Fig. 2). The GEE model, adjusted for time and nalbuphine dose, was constructed to assess the effect of group on the static and dynamic VAS. The static and dynamic VAS were lower in the ketorolac group compared to the ibuprofen group by 0.31 (95% confidence interval: 0.13 to 0.50, *P*-value 0.001) and 0.28 (95% confidence interval: 0.08 to 0.44, *P*-value 0.005), respectively.

**Table 2:** Perioperative analgesic consumption. Data are presented as median (quartiles) and frequency (%)

	Ibuprofen group (*n* = 53)	Ketorolac group (*n* = 53)	*P*-value
Intraoperative fentanyl consumption (mcg)	100 (50, 150)	50 (0, 75)	< 0.001
Time to first nalbuphine request (min)	30 (30, 30)	120 (30, 180)	< 0.001
No. of patients needing nalbuphine	53 (100%)	49 (93%)	0.118
Total nalbuphine consumption (mg)	30 (14, 40)	15 (9, 20)	< 0.001
Time to independent movement (h)	6 (6, 6)	6 (4, 6)	< 0.001

The intra- and postoperative opioid consumption was lower in the ketorolac group than that in the ibuprofen group. The time to independent movement was shorter in the ketorolac group than that in the ibuprofen group (Table 2).

The incidence of postoperative nausea and vomiting was lower in the ketorolac group than the ibuprofen group. More patients in the ketorolac group passed flatus during the first 24 h postoperatively than the ibuprofen group. On the other hand, the level of postoperative sedation according to the modified RSS was comparable between the two groups. None of the included patients developed itching or respiratory depression. Patients in the ketorolac group had a higher level of satisfaction than patients in the ibuprofen group (Table 3).

**Table 3:** Other postoperative outcomes. Data are presented as frequency (%) and median (quartiles)

	Ibuprofen group (*n* = 53)	Ketorolac group (*n* = 53)	*P*-value
Postoperative nausea	46 (87%)	22 (42%)	< 0.001
Postoperative vomiting	34 (64%)	7 (13%)	< 0.001
Passed flatus in the first 24 h	41 (77%)	53 (100%)	< 0.001
Postoperative modified RSS			
0.5 h	3 (3, 3)	3 (3, 4)	0.092
2 h	3 (2, 3)	3 (2, 3)	0.260
4 h	2 (2, 2)	2 (1, 2)	0.147
6 h	1 (1, 2)	1 (1, 2)	0.169
10 h	1 (1, 2)	1 (1, 2)	0.157
18 h	1 (1, 2)	1 (1, 1)	0.280
24 h	1 (1, 2)	1 (1, 1)	0.280
Patient’s satisfaction	5 (5, 6)	7 (6, 7)	< 0.001

*RSS*, Ramsay Sedation Score

Postoperative systolic blood pressure and heart rate were generally lower in the ketorolac group than the ibuprofen group. The intra- and postoperative systolic blood pressure and heart rate data are presented as a Supplementary file.

## Discussion

In this study, we compared the analgesic efficacy of intravenous ibuprofen and ketorolac in bariatric surgery and found that ketorolac was superior to ibuprofen. Ketorolac reduced the intra- and postoperative opioid consumption and opioid-related complications, namely the postoperative nausea and vomiting. Furthermore, patients’ satisfaction was higher in patients receiving ketorolac.

Both ketorolac and ibuprofen produce their analgesic effects through nonselective inhibition of cyclooxygenase (COX) enzymes (COX-1 and COX-2) and subsequently reduction of prostaglandin production [10]. Inhibition of COX-1 is responsible for the side effect manifestations, while inhibition of COX-2 enzyme is responsible for the analgesic anti-inflammatory effect. The relative inhibition of COX-1 and COX-2 is 330:1 in ketorolac and 2.5:1 in ibuprofen. Therefore, the risk of side effects is believed to be low during ibuprofen use [11]. On the other hand, the relative analgesic efficacy of both drugs is not well defined, and comparative reports of both drugs in perioperative setting showed conflicting results [12–15].

In line with our finding, ketorolac was superior to ibuprofen in laparoscopic cholecystectomy surgery [12]. While in urological and gynecological procedures [13, 14], both drugs showed similar analgesic profile. On the other hand, in knee arthroscopy, Uribe et al. [15] reported that ibuprofen had better analgesic profile compared to ketorolac. Based on previous data and our findings, the analgesic efficacy of both drugs could differ according to the procedure; it seems that ketorolac is superior to ibuprofen in upper abdominal and laparoscopic procedures (bariatric surgery in our study and cholecystectomy in Lee et al. study [12]). Neither of the two drugs was superior in lower abdominal surgeries [13, 14]. However, more studies are needed in different surgical settings to confirm this observation.

NSAIDs are extensively used for pain control in various settings due to their numerous advantages over other analgesic modalities [16]. NSAIDs do not produce respiratory depression nor airway obstruction which are the major limitations of opioid drugs [16]. NSAIDs produce effective analgesia for visceral pain which is not covered by abdominal field blocks [17] and do not require advanced ultrasound skills. Finally, NSAIDs do not produce cardiovascular depression which is common with neuraxial blocks. Therefore, the use of NSAIDs is a basic component of many multimodal analgesia protocols in different surgical procedures including bariatric surgery [18, 19].

Bariatric surgery is increasing in popularity in the last decade for being an effective line for management of obesity. There is increased interest in enhanced recovery programs for having early ambulation and short hospital stay after bariatric surgery. Effective pain management is a fundamental component of these programs [18, 20]. However, pain management modalities in patients with obesity are limited. Obesity is commonly associated with obstructive sleep apnea which precludes the use of opioid drugs [21, 22]. Regional techniques are relatively difficult due to anatomical problems and do not cover visceral pain. Therefore, NSAIDs are essential in bariatric surgery for being simple and effective and devoid of upper airway complications [21]. Our results suggest that ketorolac is more efficient in these patients than ibuprofen.

This study had several strengths, such as the adequate sample size and the randomized controlled double-blinded design. There are some limitations, such as being performed in a single center, using only one dose for each drug, and the absence of regional analgesic techniques. We excluded patients with obstructive sleep apnea, high STOP-Bang score, or baseline SpO2 < 95% since the pain management protocol in this study included the administration of long-acting opioids. The use of opioids in such patients increases their risk for postoperative adverse respiratory events [22].

In conclusion, in patients with obesity undergoing bariatric surgery, perioperative administration of ketorolac provided improved pain control, reduced opioid consumption, and lowered the risk of postoperative nausea and vomiting, compared to ibuprofen. Additionally, patients reported higher satisfaction with ketorolac.

## Supplementary Information

Below is the link to the electronic supplementary material.

Supplementary file1 (DOCX 62 KB)

## Data Availability

The datasets used and/or analyzed during the current study are available from the corresponding author on reasonable request.
