# Supplementary material for: Intravenous Ibuprofen Versus Ketorolac for Perioperative Pain Control in Patients with Morbid Obesity Undergoing Bariatric Surgery: A Randomized Controlled Trial
Source: Obes Surg. 2025 Mar 10;35(4):1350–6. doi: 10.1007/s11695-025-07752-5 (PMC11976341; doi:10.1007/s11695-025-07752-5)
Supplement: Supplementary file 1 — Supplementary file1 (DOCX 62 KB) [file 11695_2025_7752_MOESM1_ESM.docx]

**Supplementary figures**


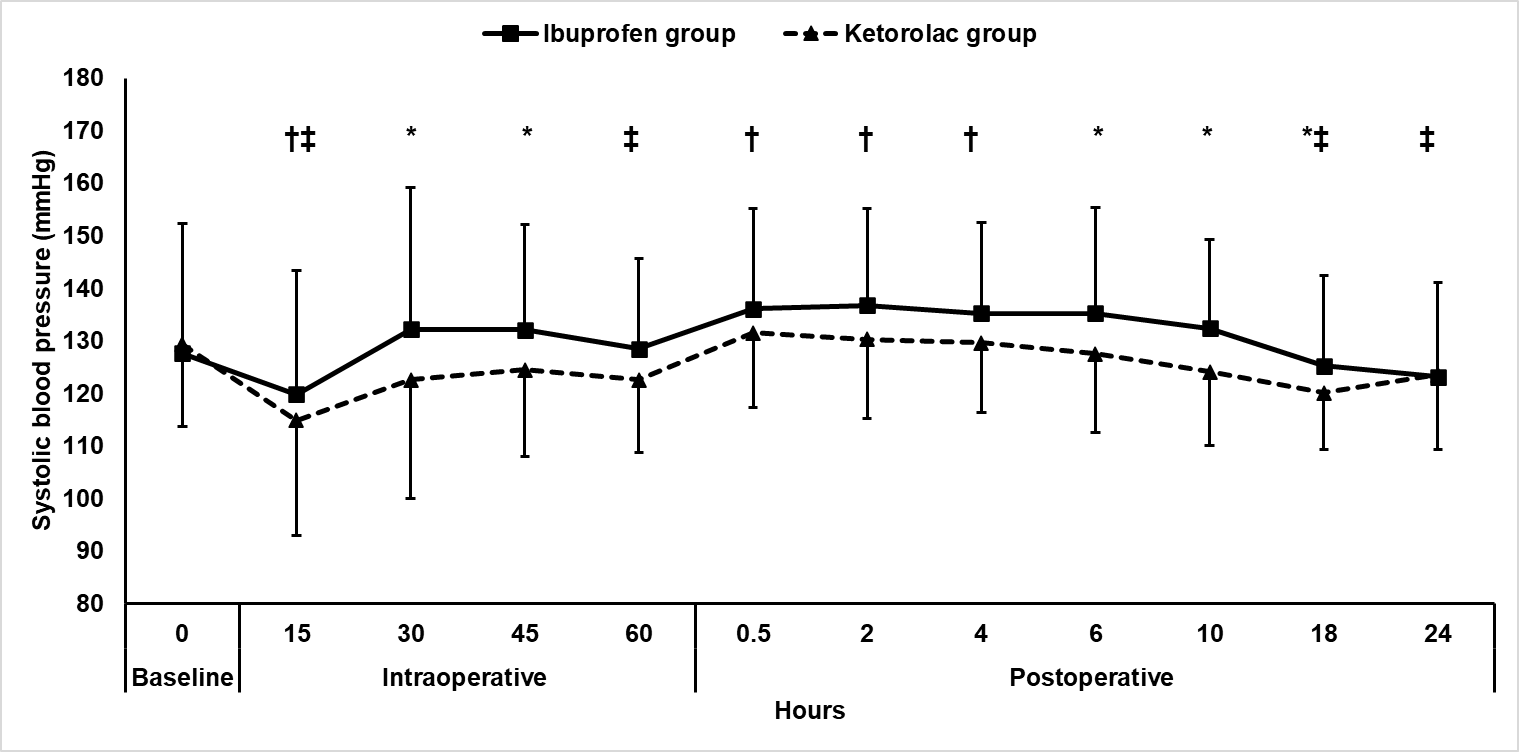


Supplementary figure 1: Systolic blood pressure, Markers represent the mean and error bars represent the standard deviation. * Denotes significance between the groups, †denotes significance in relation to baseline measurement in the Ibuprofen group, ‡denotes significance in relation to baseline measurement in the ketorolac group


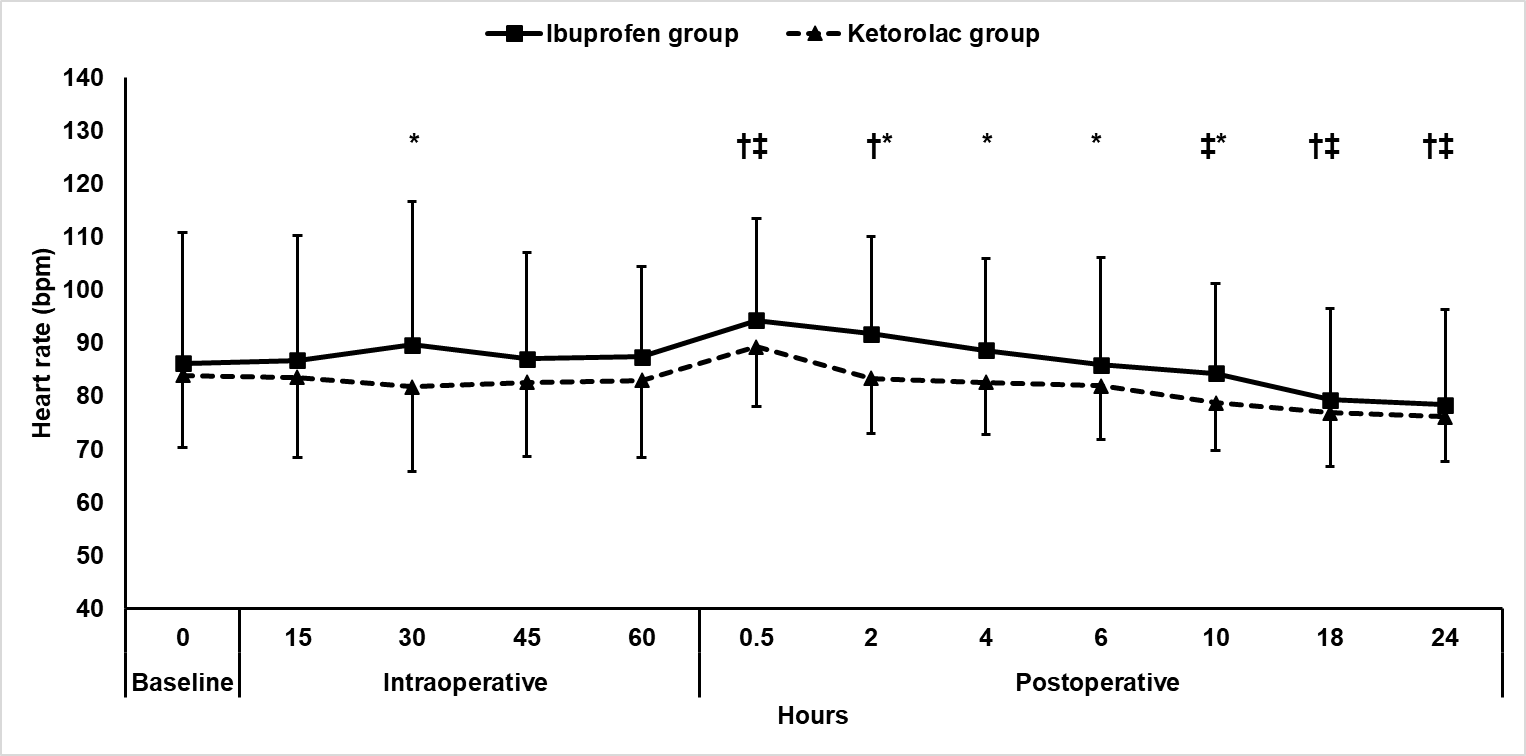


Supplementary figure 2: Heart rate. Markers represent the mean and error bars represent the standard deviation. * Denotes significance between the groups, †denotes significance in relation to baseline measurement in the Ibuprofen group, ‡denotes significance in relation to baseline measurement in the ketorolac group
